# Owner-Perceived Undesirable Behaviours in Young Dogs and Changes with Age

**DOI:** 10.3390/ani15081163

**Published:** 2025-04-17

**Authors:** Rachel H. Kinsman, Rachel A. Casey, Séverine Tasker, Ben Cooper, Kassandra Giragosian, Naomi D. Harvey, Sara C. Owczarczak-Garstecka, Lauren Samet, Jane K. Murray

**Affiliations:** 1 Dogs Trust, London EC1V 7RQ, UK; 2 Bristol Veterinary School, University of Bristol, Bristol BS40 5DU, UK; 3 Mars Veterinary Health, Shirley, Solihull B90 4BN, UK

**Keywords:** dog, puppy, undesirable behaviour, behaviour problems, behaviour, welfare, human–animal interactions, barking, recall issues, pulling on the lead

## Abstract

Dog behaviour that owners perceive as undesirable can compromise dog welfare, impact the owner and human–animal bond, and may result in relinquishment or euthanasia. This longitudinal study explored the type and percentage of owner-perceived undesirable behaviours in 6, 9, 12, 15 and 18-month-old dogs, and examined differences in the prevalence of four commonly reported behaviours across these timepoints. Owners reported their dog’s undesirable behaviours via free-text answers in self-administered surveys. Of the five timepoints studied, the highest percentage of dogs reported by their owners to display one or more undesirable behaviours was in the 12-month survey (42.1%, 513/1219). Barking, jumping up, pulling on the lead and recall issues were the most commonly reported behaviours across all timepoints. Binomial mixed-effects models revealed a significant difference in prevalence of barking, pulling on the lead and recall issues between the timepoints, but no significant difference in the occurrence of jumping up at people. With many dogs in this study being reported to display undesirable behaviour, it is crucial that stakeholders inform dog owners, especially first-time owners, about the behaviours they may experience from young dogs and where to seek appropriate training/behaviour advice to potentially reduce the risk of relinquishment related to behaviour.

## Simple Summary

Dog behaviour that owners perceive as undesirable can compromise dog welfare, impact the owner and the human–animal bond, and may result in dogs being relinquished or put to sleep. This study explored the behaviours owners perceived to be undesirable in young dogs and how the prevalence of these behaviours changed with age. Participants in a longitudinal study completed surveys about their dogs when aged 6-, 9-, 12-, 15- and 18-months-old, where they could detail any behaviours displayed by their dogs that they found to be undesirable. Many of the dogs in this study displayed one or more owner-perceived undesirable behaviours. Of the five timepoints studied, the highest percentage of dogs reported by their owners to display one or more undesirable behaviours was in the 12-month survey (42.1%). Across all five timepoints, barking, jumping up, pulling on the lead and recall issues were the most commonly reported undesirable behaviours. Analysis showed that the prevalence of barking, pulling on the lead and recall issues was significantly different between timepoints. Informing dog owners, especially first-time owners, of the types of behaviours that may be seen in young dogs and where to seek out appropriate training/behaviour advice could potentially reduce relinquishment related to behaviour.

## 1. Introduction

Dog behaviours that owners find undesirable have been widely documented (for examples, see refs. [1,2,3,4,5]). Undesirable behaviours can affect the welfare of the dog [6,7,8] and the wellbeing of the owner [9], impact the human–animal bond and have been reported as a common reason for the relinquishment [10,11,12,13,14] and euthanasia of otherwise physically healthy dogs [15,16,17,18]. Undesirable behaviours have also been cited as a common reason for dogs being returned to animal welfare organisations following failed adoptions [19,20,21].

In this paper, the term “undesirable” behaviour is used to describe behaviour that owners perceive as problematic or troublesome to them. Not all dog owners identify the same behaviours as undesirable because individuals’ opinions on the desirability of a behaviour varies [22,23]. Whether a behaviour is considered undesirable to an owner may be impacted by several factors. For example, the owner’s perception of the severity and nature of the behaviour and their perceived motivation for the behaviour, as well as the owner’s resilience, expectations, personality, previous dog ownership experience and circumstances [19,20,24,25,26]. Additionally, not all owners recognise what constitutes ‘normal’ dog behaviour or know how to cope with undesirable behaviours that may develop [27,28]. Some owners have little tolerance for behaviours that are considered within a dog’s normative behavioural repertoire, whereas other owners accept and manage behaviour which, in other contexts, might lead to relinquishment or euthanasia of the dog [26]. Therefore, it is important to understand what behaviours are commonly perceived as undesirable for owners so that stakeholders know how to best place interventions to support dog owners.

Aggression is one behaviour that is commonly perceived as undesirable [1,29,30,31,32,33], while other frequently reported behaviours include barking, jumping up, pulling on the lead, overexcitement, attention-seeking behaviours, inappropriate toileting, anxiety, and fearfulness [1,19,23,29,31,34,35,36,37,38,39]. Some behaviours identified by owners as undesirable may not be indicative of a welfare problem for the dog. However, owners may not recognise behaviours [26,40] that would be of concern to animal behaviourists as potential indicators of welfare issues (e.g., separation-related behaviours (SRBs)) or early indicators of potentially more serious undesirable behaviours (e.g., aggression). Furthermore, undesirable behaviours may be underreported as it has also been suggested that owners primarily seek help or advice for behaviours that affect people from outside their household and/or occur outside the house, potentially due to the behaviours being seen as socially unacceptable [23].

Unmodifiable factors, including genetics [41,42] and age [38,43], play a part in the development of undesirable behaviours. There are also modifiable factors that are thought to contribute to undesirable behaviours, including acquisition age [38], neutering [44,45,46], early life experiences [47,48,49,50,51], learning throughout life [52] and training [23,53,54,55,56,57].

This present study used data from the same cohort of dogs used in a previous study [26] which reported that 31.3% (n = 302/965) and 35.2% (n = 276/784) of 6- and 9-month-old dogs, respectively, showed one or more owner-perceived undesirable behaviours. The most frequently reported undesirable behaviours in 6-month-old dogs were jumping up at people, chewing/mouthing/nipping/play biting at hands/clothes, pulling on the lead and recall issues. The most frequently reported behaviours in 9-month-old dogs were pulling on the lead, jumping up at people, recall issues, and excessive/inappropriate barking (unrelated to people, or other animals) [26]. The study reported here expanded on that previous research [26] and used longitudinal data to explore owner-perceived undesirable behaviours in dogs aged 6, 9, 12, 15 and 18 months, and investigated whether the types of behaviour reported changed over the first 18 months of dogs’ lives. Stakeholders can use these findings to inform owners, especially first-time owners, about the behaviours that they may experience from their young dogs, how these behaviours may change as dogs age, and where to seek appropriate training/behaviour advice. The study aimed to achieve the following:

- Identify the percentage of owners within this cohort that reported undesirable behaviours in dogs aged 6-, 9-, 12-, 15- and 18-months-old;
- Describe the undesirable behaviours reported at each timepoint;
- Identify differences in the prevalence of four commonly reported behaviours—barking, jumping up at people, pulling on the lead and recall issues—across the five timepoints.

## 2. Materials and Methods

### 2.1. Study Design and Participants

This analysis used data gathered as part of a large longitudinal study of canine health and behaviour called ‘Generation Pup’ (https://generationpup.ac.uk/). The inclusion criteria for ‘Generation Pup’ were as follows: participants must (1) be resident in the United Kingdom (UK) or the Republic of Ireland (ROI), (2) be at least 16-years-old and (3) own a puppy under 16-weeks-old at time of registration (or under 21 weeks if the puppy had been through quarantine). The recruitment methods and study methodology have been previously described [58].

### 2.2. Data Collection

Upon registration to ‘Generation Pup’, participating dog owners are issued optional surveys when their dog reaches specific ages, which are only available for a limited period to reduce recall bias [58]. For this analysis, data were collected from five optional surveys that were issued to owners when their dogs were 6-, 9-, 12-, 15- and 18-months-old. The surveys were self-administered and completed online or via postal surveys, depending on the owner’s preference. The surveys included a range of open-ended and closed questions that addressed various aspects of health and behaviour that may impact dog welfare. Prior to analysis, all data were pseudonymised. Data were collected between May 2016 and March 2020. To remove any potential impact of the COVID-19 lockdown, surveys completed after 23 March 2020 (when restrictions around the “Stay at Home” message were put in place by the UK Government [59]) were not included in the analysis. These restrictions may have impacted the amount of time owners spent around their dogs, the owners’ perceptions of their dog’s behaviours and the behaviours of the dogs themselves.

### 2.3. Data Coding and Descriptive Statistics

To explore owner-perceived undesirable behaviours in dogs, owners were asked if their dog had ‘started to show behaviour(s) that they found a problem’ in the 6-month survey. In the 9-, 12-, 15- and 18-month surveys, owners were asked if their dog was ‘showing behaviour(s) that they found a problem’. In all surveys, if the response was ‘yes’, a free-text box was provided for the owner to ‘describe the behaviour(s) they found to be a problem’.

After familiarisation with the free-text responses, a list of undesirable behaviours (Table 1) was created in consultation with experts (one Certified Clinical Animal Behaviourist with the Association for the Study of Animal Behaviour, one with a Master of Science in clinical animal behaviour, one with a Master of Research in Animal Behaviour, and two with a Doctor of Philosophy in applied dog behaviour). The motivations for behaviours reported was not explored as part of this study. The owners’ free-text responses were then initially coded in Excel by three researchers (K.G., R.K. and L.S.), who each coded approximately 1/3 of the data, and the presence/absence of each of the undesirable behaviours in Table 1 was recorded for each response. Approximately 5% of the free-text responses were coded by more than one researcher to enable inter-rater reliability to be assessed and reported. To assess the degree to which coders consistently assigned the presence/absence of behaviours to dogs, an inter-rater reliability (IRR) analysis was preformed using R 4.2.2 [60], the R packages “irr” (v0.84.1; [61]) and “irrCAC” (v1.0; [62]). Gwet’s AC1 for each coder pair were computed then averaged to provide a single index of IRR [63]. Based on the guidelines from Landis and Koch [64], all behaviours had “almost perfect” agreement, based on their Gwet’s AC1 of >0.81 (Table A1 in Appendix A). *p*-values for the Gwet’s AC1 values were all <0.05, indicating agreement between raters was higher than expected by chance for all behaviours.

Where possible, the behaviour reported by the owner was designated a behaviour category based on the wording used by the owner (i.e., literal interpretation and no speculation/conjecture from the coders). Behaviours were not assigned emotions by coders (for example, classified as aggressive, fearful and so on). However, some owners, when reporting the behaviour deemed to be problematic, provided what they perceived to be the emotion or context of the behaviour. For example, “she is nervous around people” or “shows aggressive behaviour”. In some of these cases, the specific behaviours displayed were not provided by the owners, but in order to not lose useful detail, text such as this was categorised as ‘Displaying fear’, ‘Displaying agonistic behaviours’ and ‘Displaying reactive behaviours’ (Table 1). Additionally, a category was needed for ‘Separation-related behaviours’ for when owners reported that their dog displayed a behaviour when left alone or when the owner stated “Separation-related behaviours” but did not detail the specific behaviours shown. These categories were only used when the owner stated the terms listed in Table 1. Contact behaviours (which included biting, play-biting, nipping, snapping, mouthing, grabbing clothing, and grabbing) were coded together during the initial coding process of the three researchers. This was also the case with destructive behaviours (which included chewing, destroying furniture/objects not belonging to the dog and digging). Any behaviours that could not be coded into an existing category by the three researchers were coded as ‘Other’, as the frequencies of these behaviours were too low to justify reporting. Therefore, across the five surveys, 1.7–2.7% of ‘Other’ behaviours were not coded.

The number of owner-perceived problem behaviours per dog at each timepoint was calculated by summing the number of behaviours shown that could be coded into one of the behaviours listed in Table 1. For some of the behaviours listed in Table 1 (barking, jumping up, contact behaviours, growling and chasing), some owners had provided information regarding the perceived target of the behaviour. To avoid losing the owner-perceived targets of the behaviour, the additional details were coded by one researcher (RK). Also, for the behaviour ‘eating non-food items’, some owners stated what items were eaten, and the sub-categories of ‘faeces’ and ‘other items’ were used. To facilitate more detailed analysis regarding the owner-reported perceived targets of contact behaviours, the behaviours coded as contact behaviours (detailed in Table 1) were assigned one of the seven separate categories by one researcher (R.K.).

### 2.4. Sample Size and Power Analysis

The sample size was dependent on the number of owners who completed the three mandatory surveys and answered questions about whether their dog showed undesirable behaviours in one or more of the 6-, 9-, 12-, 15- and/or 18-month surveys. All available data were used, and owners did not need to have completed all timepoint surveys to be included in the analysis. Additionally, although owners could register up to five puppies with ‘Generation Pup’, to reduce any impacts of clustering at the household level, one puppy per household was randomly selected (using https://randomizer.org/) for inclusion in the dataset. As recruitment to ‘Generation Pup’ was still ongoing at the time of data collection, the number of completed surveys decreased for each successive survey because not all dogs included in this study had reached the age of issue for the later surveys (Table 2). Additionally, loss to follow-up will have contributed to the reduction in sample size over time in ‘Generation Pup’; this has been detailed elsewhere [58].

A post hoc power analysis was carried out to determine the minimum effect size that could be robustly detected in our sample using our binomial mixed-effects models. Datasets of 460 individuals were simulated, as this represented the typical sample size used in our analysis, where data were available for all individuals. Data were simulated over a range of differences in odds, and for each simulated individual, a random intercept was also drawn from a normal distribution. Binomial mixed-effects models were then fitted to these simulated data, with a structure identical to those used with our real data. For a given effect size, 1000 datasets were simulated and the proportion of these in which our models were able to detect a difference in odds were recorded. Using this process, a minimum detectable effect size at the 80% power level of 0.6 was determined, corresponding to a fold change in odds of 1.82× or 0.55×.

### 2.5. Statistical Analysis

After four of the most common owner-perceived undesirable behaviours across the five timepoints were identified, a statistical analysis was performed to assess differences in the prevalence of these behaviours across the five timepoints. Four binomial mixed-effects models, with individual identity as a random intercept, were fitted using the R package “lme4” (v1.1-31; [65]). Post hoc Tukey’s Honestly Significant Difference tests for multiple comparisons were performed using the R package “multcomp” (v1.4-22; [66]). The response variable for each model was the behaviour (y/n), and the fixed effect was survey timepoint (6, 9, 12, 15 and 18 months). *p*-values and confidence intervals were calculated using the R packages "lmerTest” (v; 3.1-3; [67]) and “ciTools” (v 0.6.1; [68]), respectively. Model assumptions of the normality and equal variance of the residuals were confirmed by visual inspection and using the R package “DHARMa” (v0.4.7; [69]). All analyses were performed using R 4.2.2 [60].

## 3. Results

Table 2 utilises all survey data available and summarises the percentage of dogs reported to display one or more undesirable behaviours at each timepoint. The highest percentage of owner-reported undesirable behaviours was recorded in the 12-month survey (42.1%, n = 513/1219, Table 2).

Table 3 summarises the owner-perceived undesirable behaviours that were reported at each timepoint and utilises all available data. Barking was the most commonly reported undesirable behaviour at each timepoint, as 7.9% (n = 136/1718), 9.9% (n = 144/1451), 13.4% (n = 163/1219), 11.9% (n = 98/823) and 10.5% (n = 72/684) of owners reported this behaviour in the 6-, 9-, 12-, 15- and 18-month surveys, respectively (Table 3).

**Figure 1:**
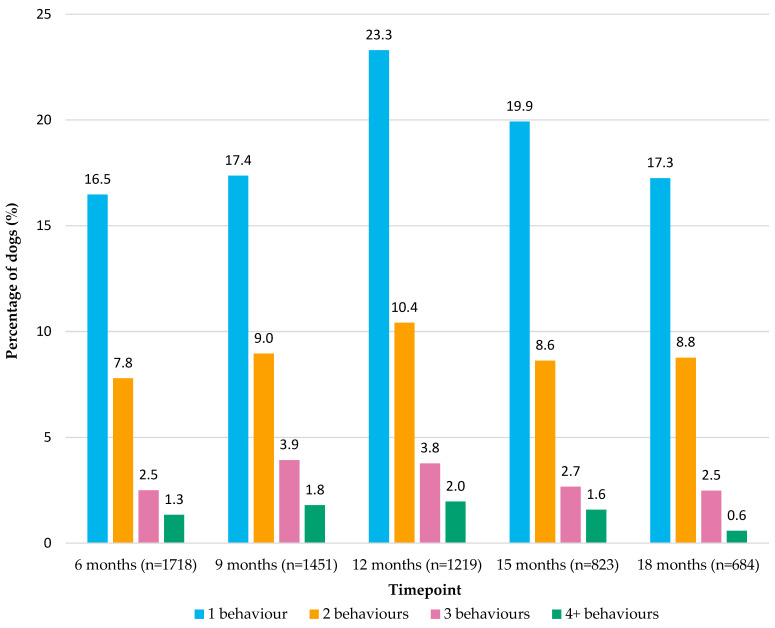
Frequency of the number of owner-perceived undesirable behaviours in Table 1 reported for each dog aged 6-, 9-, 12-, 15- and 18-months-old for those dogs reported to show one or more undesirable behaviours.

Figure 1 summarises the number of behaviours listed in Table 1 that each dog was reported to show at each of the five timepoints. Of the dogs who were reported to show one or more undesirable behaviours, most commonly, only one behaviour was reported, and this was consistent for all five timepoints (Figure 1). The median number of owner-perceived undesirable behaviours, where an undesirable behaviour was reported, was one per dog across all five timepoints. The range was 1–7 behaviours reported for 6- and 9-month-old dogs, and the range was 1–6 behaviours for 12-, 15- and 18-month-old dogs. Finally, Table 4 summarises the owner-perceived targets of some of the undesirable behaviours listed in Table 1 for dogs aged 6-, 9-, 12-, 15- and 18-months-old. For example, barking was commonly perceived as directed at people as opposed to other dogs, animals, noises and so on (Table 4). Play biting, nipping and biting were most often reported as being directed towards people compared to towards other dogs (Table 4).

Binomial mixed-effects models were used to assess differences in the prevalence of the four most commonly reported behaviours, (barking, jumping up at people, pulling on the lead and recall issues), across the five timepoints (Table 5). The analysis revealed significant differences in prevalence of barking behaviour, with 12-month-old dogs being reported to bark more than 6- and 9-month-old dogs, and 15-month-old dogs barking more than 6-month-olds. Likewise, 9-month-old dogs were reported to pull on the lead significantly more often than 15-month-olds, and 12-month-old dogs pulled significantly more often than 6-, 15-, and 18-month-old dogs. Recall issues were reported significantly less often in 6-month-old and 18-month-old dogs than in 9-, 12-, and 15-month-old dogs. There was no significant difference in the occurrence of jumping up at people between the five timepoints (Table 5).

## 4. Discussion

This study explored the owner-perceived undesirable behaviours displayed by dogs aged 6-, 9-, 12-, 15- and 18-months-old that were participating in a longitudinal study. These longitudinal data offer a unique insight into owner-perceived undesirable behaviours by enabling investigation into the percentage of dogs reported to have undesirable behaviours, what the behaviours were and how the percentages of certain behaviours changed as dogs aged.

At each of the five timepoints, more than half of the dogs in the cohort were reported to not display any owner-perceived undesirable behaviours, while between 29.9% and 42.1% of dogs were reported to display one or more undesirable behaviours (Table 1). Previously documented figures on the percentage of dogs displaying owner-perceived undesirable behaviour range from 40% to 87% [1,2,3,4,5]. The findings reported here are lower or, in the case of the 12-month survey data, at the lower end of this range. This may relate to differences between study populations, such as the age groups of dogs sampled, changes or differences in social and/or cultural tolerance regarding what undesirable behaviours are [3,16,19,34,36] or differences in the data collection techniques (discussed later in this section).

In this study, the median number of undesirable behaviours (displayed by dogs showing one or more undesirable behaviours) was one, and this was consistent for all five timepoints, and also consistent with the results reported from a smaller ‘Generation Pup’ dataset by Lord et al. [26]. A previous study by Dinwoodie et al. reported a median of three undesirable behaviours in dogs under 3-years-old [5]. The difference in the median number of behaviours reported is speculated to be due to some of the factors listed in the previous paragraph. Additionally, the ‘Generation Pup’ study recruited puppies under 16-weeks-old, whereas, in contrast, 43% of dogs in the study by Dinwoodie et al. (2019) [5] were acquired from rehoming centres, suggesting the possibility of less stability/consistency in their life experiences, which may have led to increased fearfulness [52].

The most commonly reported behaviours at each of the timepoints were barking, jumping up, pulling on the lead and recall issues (Table 3). This is in agreement with other reports documenting common undesirable behaviours in survey-based studies [23,26,34,38,39]. Barking was the most reported undesirable behaviour in the current study—ranging between 7.9% and 13.4% (Table 3). Previous studies have reported similar or slightly higher percentages (2.7–18%) [1,5,31] and noted barking as being more common in juveniles as opposed to in puppies or adults [19]. Jumping up was reported in 5.2% to 7.9% of the dogs (Table 3), and of dogs that were reported to jump up, most were reported to jump up at people. In a similarly aged dog population (also using pre-pandemic data), 30.3% of 915 dogs were reported to jump up at people [39], which is much higher than the figures reported in our cohort (Table 4). A possible explanation for this is that not all owners in our study perceived jumping up to be an undesirable behaviour and hence did not report it as such. Additionally, some owners reported ‘jumping up’ but did not specify the target (for example, people, dogs, surfaces); therefore, these cases were classified as jumping up at an ‘unspecified target’. It is possible that this led to jumping up at people being underreported within the cohort, as these owners may have intended ‘jumping up’ to be interpreted as jumping up at people, but this was unknown and therefore could not be coded as such.

Although the percentage of dogs reported to display contact behaviours (as a combined category) was relatively high in comparison to other behaviours across the five timepoints (Table 3), when the individual behaviours that made up the contact behaviours category were considered separately (Table 4), the individual percentages were not as high as the four most-reported behaviours. The words owners used to describe their dogs’ contact behaviour defined how the behaviour was coded (Table 1). Clear definitions of these behaviours or the severity (e.g., whether medical care was needed) were not given to or by owners. Thus, the same type of behaviour might be reported as mouthing by one owner and as biting by another. Additionally, as “biting” can be viewed as a negative behaviour, owners may be inclined to under-report or play down the behaviour according to the terminology used. The percentage of dogs reported to “bite” people ranged from 0.6% to 2.0%, with the highest percentage being reported for 6-month-old dogs. A greater understanding and careful consideration of the common definitions of bite, mouthing and other contact behaviours is needed [70,71], and providing survey participants with definitions would be beneficial to avoid under- or over-reporting of certain behaviours.

Despite aggression being one of the most commonly reported undesirable behaviours within canine behaviour [1,29,30,31,32,33], very few owners in the ‘Generation Pup’ cohort used terminology that indicated they thought their dog was aggressive. The percentage of dogs reported to display agonistic behaviours (i.e., if the owner used the term ‘aggressive’ and/ or ‘aggression’ in their text) was lowest at 6 months (0.9%) and this increased gradually as the dogs aged, with the highest percentage being reported in the 18-month survey (3.4%). There is the potential that the percentages of agonistic behaviours could in fact be higher as owners may not recognise the behaviours [26,40] or may be inclined not to report such behaviours due to social desirability bias. The authors speculate that as dogs age, any agonistic behaviours may become more established and/or difficult to manage, potentially leading to increased reporting.

The statistical analysis showed significant differences across the five timepoints in the prevalence of barking, pulling on the lead, and recall issues, but not in the percentage of dogs that were reported to jump up at people. Reports of barking, pulling on the lead, recall issues and jumping up at people all peaked in 12-month-old dogs and declined thereafter. Although influenced by breed, for most dogs the pubertal period occurs between 6 months and 1 year of age) [72]. Dogs aged between 1 and 2 years are considered post-pubertal and are normally fully grown, but typically still experience some adolescent development (the final stage of reproductive maturation) [72]. It has previously been documented that adolescence in dogs can be accompanied by adolescent-phase behaviour—a passing phase of carer-specific conflict-like behaviours (including reduced responsiveness to commands and trainability [73]), which may help to explain why the behaviours peaked in dogs aged 12 months and subsequently declined. This is potentially an important message to share with owners of young dogs, as although undesirable behaviours may increase around puberty, these behaviours could improve again as the dog continues to age. However, it is unknown whether the observed declines occurred as part of the dog’s maturation or were due to the behaviours (such as pulling on the lead and recall issues) being more amenable to training and owners taking steps to address the behaviours they perceived as undesirable. Alternatively, due to the method of data collection, owners may simply have not perceived those behaviours as being undesirable by the subsequent survey timepoint(s) or perhaps had accepted or adapted to the behaviour.

The findings of this research may differ from previous studies for a variety of reasons. For example, some of the previous studies reporting a higher prevalence of undesirable behaviours included dogs of all ages. Existing research has shown that a dog’s behaviour can be influenced by age. Cognitive function can deteriorate as dogs get older, and this can manifest in various ways, including forgetting previously learned behaviours, developing new fears, and experiencing a decline in memory and learning ability [43]. Also, owners’ perceptions of their dogs’ behaviour can change over time [74]. Furthermore, the previously mentioned studies were based in other countries (e.g., America and Denmark), and it is plausible that as dog legislation and socially acceptable behaviours vary between countries, dog owners’ opinions of what constitutes undesirable behaviours may also differ. Additionally, variation between the methodologies and participants of the studies must be considered. In this analysis, the data were gathered through free-text responses rather than multiple choice questions. This approach might have influenced both the type and frequency of undesirable behaviours owners reported, as a pre-defined list of suggested behaviours was not given. A free-text box also requires more effort to complete compared to selecting/ticking boxes. It is speculated that owners may have only reported problems that immediately sprung to mind and/or potentially those behaviours that most affected them; hence, the prevalence of reported behaviours is anticipated to be lower in this analysis compared to studies using multiple choice questions. ‘Generation Pup’, being a longitudinal study, requires considerable participant commitment, which may attract a different type of dog owner compared to those that participate in cross-sectional studies. This may affect how representative the ‘Generation Pup’ cohort is compared to the wider dog population. Self-selection bias and under-representation of lower socioeconomic backgrounds can occur with longitudinal studies, although the effects are thought to be limited [75]. Further investigation of the dataset, including association between owner characteristics and owner-reported dog behaviour, would be required to establish whether the profile of the cohort owners is a likely contributor towards the lower prevalence of reported behaviours in comparison to other published studies. A final consideration to reiterate is that owners sometimes used specific terms such as “separation-related behaviours” and “shows aggression” without detailing the behaviours, and it cannot be confirmed whether the owners were using the term in the same way as an animal behaviourist or veterinarian would.

Within ‘Generation Pup’ surveys, owners are asked about a wide range of behavioural signs that could indicate undesirable behaviours, including how their dog responded to meeting unfamiliar dogs and people, issues with walking their dog and responses to noises. These data were not explored here, as this study focused on the free-text responses owners volunteered about undesirable behaviours

This study offers new insights into age-specific owner-perceived undesirable behaviours in young dogs, as three of the well-cited studies in this area [1,2,3] are over 25 years old. The internet and social media now provide easy access to information (although the quality and evidence base of this information can vary considerably). In the UK, organisations offering dog training classes and puppy schools are now commonplace and more accessible. These factors have potentially affected how owners view ownership, dog behaviour and training techniques. The findings presented here could help owners to manage their expectations of their dogs’ behaviour. Additionally, welfare organisations and other stakeholders can use these findings to help inform the support that they provide owners regarding how to minimise, prevent and address specific behaviours (such as barking, jumping up, pulling on the lead, recall issues and contact behaviours), particularly prior to 12 months of age. As jumping up, pulling on the lead and recall issues can be considered training issues, emphasising the importance of training and the potential training difficulties that may occur during adolescence may also help owners. The findings presented on the overall prevalence of owner-perceived undesirable behaviours can equip veterinary professionals with knowledge that may increase confidence in further incorporating discussions, expectations and management regarding undesirable behaviours into consultations with clients.

The sources of help used by owners for undesirable behaviours have been explored for dogs aged 6 and 9 months in the ‘Generation Pup’ cohort, along with risk factors for owners reporting undesirable behaviours [26]. Further research within the ‘Generation Pup’ cohort will expand on owner help-seeking behaviour to include data from other timepoints. Understanding the needs of owners and their dogs allows welfare organisations and other stakeholders to better support behaviour welfare and to minimise the impact of owner-perceived undesirable behaviours on dogs, their owners and the human–animal bond.

## 5. Conclusions

This study identified that more than a quarter of young dogs in this cohort displayed one or more behaviours that their owners perceived as undesirable. This is concerning, as the study by Boyd et al. (2018) [17] reported that undesirable behaviours are a considerable risk factor of euthanasia for dogs under 3-years-old. The percentage of dogs reported to display undesirable behaviours was highest in dogs aged 12 months. Of the dogs reported to display undesirable behaviours, dogs were reported typically to display just one undesirable behaviour. The common behaviours of barking, pulling on the lead and recall issues peaked at 12 months, which coincides with adolescence and has been reported as a peak age at which dogs are relinquished [76,77].

Research in this area can help stakeholders provide owners, especially first-time owners, with evidence-based information about the behaviour they may encounter in young dogs and as their dogs age. Providing owners with information on where to seek appropriate training and/or behaviour advice, particularly during adolescence, could encourage owners to be proactive in seeking training for their dog. This may circumvent undesirable behaviours before they arise or develop further, which could lead to a reduction in dogs being relinquished to welfare organisations or euthanised due to behavioural reasons.

## Figures and Tables

**Table 1:** List of owner-perceived undesirable behaviour categories and brief descriptions that were assigned to free-text responses from owners during coding.

Behaviour	Owner Terms/Descriptions Used to Code This Behaviour
Barking	Barking
Jumping up	Jumping up
Contact behaviours	Biting; play-biting; nipping; snapping; mouthing; grabbing clothing; grabbing
Pulling on the lead	Pulling on the lead; lead pulling; pulling to get to/away from; similar words
Recall issues	Recall; not coming back when called; similar words
Destructive behaviour	Chewing and/or destroying of furniture/objects not belonging to the dog; digging
Toileting	Wetting; elimination; similar words
Separation-related behaviours	Separation related; separation anxiety; when alone; when not with owner
Eating non-food items	Described as eating particular items; coprophagia; similar words
Displaying fear	Described as fearful; anxious; wary; skittish; scared; nervous; similar words
Growling and/or snarling	Growling; snarling
Humping	Humping
Chasing	Chasing
Stealing	Stealing items or food
Resource guarding	Resource guarding; possessive; similar words
Displaying agonistic behaviours	Aggressive; aggression
Scavenging	Eating food found on the floor or in bins
Lunging	Lunging
Displaying reactive behaviours	Reactive; reacts

**Table 2:** Number of 6, 9, 12, 15 and 18-month surveys available for analysis, and the number and percentage of dogs reported to display one or more undesirable behaviours.

Survey Timepoint	Number of Surveys Available for Analysis	Percentage of Dogs with ≥1 Undesirable Behaviours [95% Confidence Interval]
6 months	1718	29.9 [27.7–32.1]
9 months	1451	34.0 [31.6–36.5]
12 months	1219	42.1 [39.3–44.9]
15 months	823	35.1 [31.9–38.4]
18 months	684	32.0 [28.6–35.6]

**Table 3:** Owner-perceived undesirable behaviours in dogs reported in the 6, 9, 12, 15 and 18-month surveys.

	Number of Owner Reports at:				
Undesirable Behaviour Reported	6 Months (n = 1718) *n* (%)	9 Months (n = 1451) *n* (%)	12 Months (n = 1219) *n* (%)	15 Months (n = 823) *n* (%)	18 Months (n = 684) *n* (%)
Barking	136 (7.9)	144 (9.9)	163 (13.4)	98 (11.9)	72 (10.5)
Jumping up	90 (5.2)	114 (7.9)	90 (7.4)	49 (6.0)	38 (5.6)
Contact behaviours ^a^	88 (5.1)	72 (5)	63 (5.2)	27 (3.3)	24 (3.5)
Pulling on the lead	66 (3.8)	79 (5.4)	81 (6.6)	22 (2.7)	23 (3.4)
Recall issues	57 (3.3)	73 (5.0)	80 (6.6)	48 (5.8)	19 (2.8)
Destructive behaviour ^b^	54 (3.1)	50 (3.4)	40 (3.3)	17 (2.1)	9 (1.3)
Toileting	40 (2.3)	23 (1.6)	17 (1.4)	6 (0.7)	5 (0.7)
Separation-related behaviours	40 (2.3)	27 (1.9)	35 (2.9)	12 (1.5)	8 (1.2)
Eating non-food items	27 (1.6)	22 (1.5)	17 (1.4)	11 (1.3)	5 (0.7)
Displaying fear	31 (1.8)	43 (3.0)	48 (3.9)	31 (3.8)	22 (3.2)
Growling and/or snarling	25 (1.5)	20 (1.4)	26 (2.1)	15 (1.8)	10 (1.5)
Humping	22 (1.3)	13 (0.9)	12 (1.0)	5 (0.6)	1 (0.1)
Chasing	20 (1.2)	33 (2.3)	28 (2.3)	21 (2.6)	17 (2.5)
Stealing	19 (1.1)	18 (1.2)	13 (1.1)	12 (1.5)	4 (0.6)
Resource guarding	18 (1.0)	11 (0.8)	15 (1.2)	8 (1.0)	6 (0.9)
Displaying agonistic behaviours	16 (0.9)	25 (1.7)	24 (2.0)	18 (2.2)	23 (3.4)
Scavenging	16 (0.9)	5 (0.3)	4 (0.3)	6 (0.7)	3 (0.4)
Lunging	8 (0.5)	17 (1.2)	14 (1.1)	8 (1.0)	6 (0.9)
Displaying reactive behaviours	8 (0.5)	10 (0.7)	14 (1.1)	11 (1.3)	13 (1.9)

^a^ Contact behaviour included biting, play-biting, nipping, snapping, mouthing, grabbing clothing and grabbing. ^b^ Destructive behaviour included chewing and/or destroying furniture/objects not belonging to the dog and digging.

**Table 4:** Owner-perceived targets of undesirable behaviours in dogs aged 6-, 9-, 12-, 15- and 18-months-old. Owners could report >1 target for a dog.

	Number of Owner Reports at:				
Undesirable Behaviours Reported	6 Months (n = 1718) *n* (%)	9 Months (n = 1451) *n* (%)	12 Months (n = 1219) *n* (%)	15 Months (n = 823) *n* (%)	18 Months (n = 684) *n* (%)
**Barking at (all)**	136 (7.9)	144 (9.9)	163 (13.4)	98 (11.9)	72 (10.5)
**People (all)**	42 (2.4)	44 (3.0)	63 (5.2)	46 (5.6)	27 (3.9)
Household people	15 (0.9)	25 (1.7)	23 (1.9)	9 (1.1)	8 (1.2)
Non-household people	20 (1.2)	20 (1.4)	39 (3.2)	26 (3.2)	12 (1.8)
Unspecified people	9 (0.5)	7 (0.5)	8 (0.7)	11 (1.3)	8 (1.2)
**Dogs (all)**	29 (1.7)	30 (2.1)	42 (3.4)	36 (4.4)	26 (3.8)
At or triggered by household dogs	0 (0)	3 (0.2)	1 (0.1)	2 (0.2)	0 (0)
Non-household dogs	29 (1.7)	28 (1.9)	41 (3.4)	35 (4.3)	26 (3.8)
**Animals**	9 (0.5)	9 (0.6)	15 (1.2)	3 (0.4)	6 (0.9)
**Vehicles**	3 (0.2)	0 (0)	3 (0.2)	2 (0.2)	0 (0)
**Cyclists/pedal bikes**	2 (0.1)	2 (0.1)	2 (0.2)	1 (0.1)	1 (0.1)
**Joggers**	3 (0.2)	1 (0.1)	2 (0.2)	2 (0.2)	1 (0.1)
**People passing the house**	4 (0.2)	2 (0.1)	8 (0.7)	2 (0.2)	2 (0.3)
**When separated from owner**	9 (0.5)	6 (0.4)	12 (1)	2 (0.2)	0 (0)
**When in the car**	6 (0.3)	5 (0.3)	5 (0.4)	2 (0.2)	3 (0.4)
**Noises**	16 (0.9)	14 (1.0)	14 (1.1)	12 (1.5)	9 (1.3)
**Barking to be let out**	2 (0.1)	1 (0.1)	2 (0.2)	2 (0.2)	1 (0.1)
**Unspecified**	40 (2.3)	50 (3.4)	37 (3)	21 (2.6)	14 (2.0)
**Other**	6 (0.3)	4 (0.3)	2 (0.2)	1 (0.1)	0 (0)
**Jumping up at (all)**	90 (5.2)	114 (7.9)	90 (7.4)	49 (6.0)	38 (5.6)
**People (all)**	67 (3.9)	69 (4.8)	66 (5.4)	41 (5.0)	27 (3.9)
Household people	23 (1.3)	19 (1.3)	14 (1.1)	12 (1.5)	3 (0.4)
Non-household people	16 (0.9)	23 (1.6)	21 (1.7)	14 (1.7)	8 (1.2)
Unspecified people	38 (2.2)	32 (2.2)	35 (2.9)	18 (2.2)	16 (2.3)
**Dogs (all)**	2 (0.1)	6 (0.4)	5 (0.4)	3 (0.4)	0 (0)
Household dogs	1 (0.1)	0 (0)	0 (0)	0 (0)	0 (0)
Non-household dogs	1 (0.1)	6 (0.4)	5 (0.4)	2 (0.2)	0 (0)
**Vehicles**	2 (0.1)	1 (0.1)	1 (0.1)	0 (0)	0 (0)
**Cyclists/pedal bikes**	0 (0)	1 (0.1)	0 (0)	1 (0.1)	0 (0)
**Surfaces or furniture**	16 (0.9)	15 (1.0)	8 (0.7)	7 (0.9)	4 (0.6)
**Unspecified target**	67 (3.9)	29 (2.0)	13 (1.1)	4 (0.5)	9 (1.3)
**Biting (all)**	36 (2.1)	25 (1.7)	16 (1.3)	8 (1.0)	6 (0.9)
**People**	34 (2.0)	24 (1.7)	14 (1.1)	6 (0.7)	4 (0.6)
**Another household dog(s)**	2 (0.1)	1 (0.1)	4 (0.3)	2 (0.2)	1 (0.1)
**Non-household dog(s)**	0 (0)	0 (0)	0 (0)	0 (0)	1 (0.1)
**Play biting (all)**	9 (0.5)	4 (0.3)	5 (0.4)	2 (0.2)	0 (0)
**People**	9 (0.5)	4 (0.3)	5 (0.4)	1 (0.1)	0 (0)
**Another household dog**	1 (0.1)	0 (0)	0 (0)	1 (0.1)	0 (0)
**Nipping (all)**	16 (0.9)	19 (1.3)	12 (1.0)	9 (1.1)	6 (0.9)
**People**	14 (0.8)	17 (1.2)	11 (0.9)	9 (1.1)	5 (0.7)
**Another household dog(s)**	2 (0.1)	1 (0.1)	0 (0)	0 (0)	0 (0)
**Non-household dog(s)**	0 (0)	1 (0.1)	1 (0.1)	0 (0)	1 (0.1)
**Snapping (all)**	8 (0.5)	5 (0.3)	20 (1.6)	4 (0.5)	7 (1.0)
**People**	6 (0.3)	3 (0.2)	8 (0.7)	2 (0.2)	3 (0.4)
**Another household dog(s)**	2 (0.1)	0 (0)	3 (0.2)	1 (0.1)	0 (0)
**Non-household dog(s)**	0 (0)	1 (0.1)	9 (0.7)	1 (0.1)	4 (0.6)
**Household cats**	0 (0)	1 (0.1)	0 (0)	0 (0)	0 (0)
**Mouthing people**	27 (1.6)	25 (1.7)	14 (1.1)	6 (0.7)	2 (0.3)
**Biting/grabbing clothing**	12 (0.7)	8 (0.6)	5 (0.4)	1 (0.1)	4 (0.6)
**Grabbing**	3 (0.2)	4 (0.3)	1 (0.1)	2 (0.2)	5 (0.7)
**Growling and/or snarling (all)**	25 (1.5)	20 (1.4)	26 (2.1)	15 (1.8)	10 (1.5)
**People (all)**	12 (0.7)	8 (0.6)	9 (0.7)	6 (0.7)	2 (0.3)
Household people	9 (0.5)	3 (0.2)	5 (0.4)	3 (0.4)	1 (0.1)
Non-household people	3 (0.2)	5 (0.3)	2 (0.2)	3 (0.4)	1 (0.1)
Unspecified people	0 (0)	0 (0)	2 (0.2)	0 (0)	0 (0)
**Dogs (all)**	11 (0.6)	8 (0.6)	16 (1.3)	7 (0.9)	6 (0.9)
Household dogs	3 (0.2)	1 (0.1)	5 (0.4)	0 (0)	1 (0.1)
Non-household dogs	7 (0.4)	7 (0.5)	11 (0.9)	7 (0.9)	5 (0.7)
Unspecified dogs	1 (0.1)	0 (0)	1 (0.1)	0 (0)	0 (0)
**Household cats**	0 (0)	1 (0.1)	1 (0.1)	1 (0.1)	1 (0.1)
**Unspecified target**	4 (0.2)	3 (0.2)	0 (0)	0 (0)	0 (0)
**Other ^a^**	8 (0.5)	0 (0)	1 (0.1)	1 (0.1)	0 (0)
**Chasing (all)**	20 (1.2)	33 (2.3)	28 (2.3)	21 (2.6)	17 (2.5)
**Wildlife/livestock**	7 (0.4)	16 (1.1)	14 (1.1)	11 (1.3)	6 (0.9)
**Dogs**	2 (0.1)	3 (0.2)	6 (0.5)	2 (0.2)	1 (0.1)
**Cats**	4 (0.2)	9 (0.6)	9 (0.7)	3 (0.4)	3 (0.4)
**Vehicles**	6 (0.3)	4 (0.3)	4 (0.3)	1 (0.1)	0 (0)
**Cyclists/pedal bikes**	1 (0.1)	5 (0.3)	1 (0.1)	5 (0.6)	5 (0.7)
**Joggers**	1 (0.1)	3 (0.2)	1 (0.1)	5 (0.6)	3 (0.4)
**People (incl. children)**	0 (0)	0 (0)	1 (0.1)	0 (0)	1 (0.1)
**Other ^b^**	2 (0.1)	1 (0.1)	0 (0)	0 (0)	2 (0.3)
**Eating non-food items (all)**	27 (1.6)	22 (1.5)	17 (1.4)	11 (1.3)	5 (0.7)
**Faeces**	15 (0.9)	14 (1.0)	9 (0.7)	6 (0.7)	5 (0.7)
**Other items ^c^**	14 (0.8)	11 (0.8)	11 (0.9)	5 (0.6)	2 (0.3)

^a^ Other growling/snarling responses included at the vacuum cleaner and at buses. ^b^ Other chasing responses included balls, shadow, light and reflections, and one owner did not specify. ^c^ Other items included fabrics, stones, soil, vomit, flowers, sticks and unspecified items.

**Table 5:** Pairwise comparisons for the binomial regression models for the prevalence of barking, jumping up at people, pulling on the lead and recall issues across the five timepoints. Post hoc analysis used Tukey’s Honestly Significant Difference test for multiple comparisons. Logistic regression coefficients were exponentiated and are presented as multiplicative effects.

Undesirable Behaviour	Comparisons	Model Estimate	95% CI		*p*
			Lower	Upper	
Barking	6 and 9 months	1.36	0.79	2.33	0.522
	6 and 12 months	3.04	1.78	5.20	<0.001
	6 and 15 months	2.37	1.30	4.31	<0.001
	6 and 18 months	1.74	0.90	3.36	0.148
	9 and 12 months	2.24	1.32	3.79	<0.001
	9 and 15 months	1.74	0.96	3.15	0.080
	9 and 18 months	1.28	0.67	2.46	0.843
	12 and 15 months	0.78	0.44	1.38	0.752
	12 and 18 months	0.57	0.30	1.08	0.115
	15 and 18 months	0.73	0.37	1.45	0.727
Jumping up at people	6 and 9 months	1.50	0.71	3.16	0.575
	6 and 12 months	1.73	0.80	3.73	0.289
	6 and 15 months	1.48	0.63	3.51	0.720
	6 and 18 months	0.92	0.36	2.37	0.999
	9 and 12 months	1.16	0.55	2.43	0.984
	9 and 15 months	0.99	0.43	2.30	1.000
	9 and 18 months	0.61	0.24	1.55	0.604
	12 and 15 months	0.86	0.37	1.99	0.987
	12 and 18 months	0.53	0.21	1.34	0.339
	15 and 18 months	0.62	0.23	1.67	0.683
Pulling on the lead	6 and 9 months	1.95	0.96	3.92	0.073
	6 and 12 months	2.61	1.28	5.34	0.002
	6 and 15 months	0.54	0.21	1.39	0.378
	6 and 18 months	0.84	0.33	2.15	0.985
	9 and 12 months	1.34	0.69	2.63	0.752
	9 and 15 months	0.28	0.11	0.70	0.001
	9 and 18 months	0.43	0.17	1.07	0.086
	12 and 15 months	0.21	0.08	0.52	<0.001
	12 and 18 months	0.32	0.13	0.80	0.006
	15 and 18 months	1.56	0.52	4.64	0.799
Recall issues	6 and 9 months	2.53	1.22	5.25	0.005
	6 and 12 months	2.95	1.42	6.13	<0.001
	6 and 15 months	2.92	1.29	6.57	0.003
	6 and 18 months	0.84	0.31	2.26	0.989
	9 and 12 months	1.17	0.59	2.29	0.971
	9 and 15 months	1.15	0.54	2.45	0.986
	9 and 18 months	0.33	0.13	0.86	0.014
	12 and 15 months	0.99	0.47	2.1	1.000
	12 and 18 months	0.28	0.11	0.73	0.003
	15 and 18 months	0.29	0.11	0.78	0.006

## Data Availability

The data are not publicly available due to the ethical approval of participant informed consent that included Generation Pup participants being informed that we will remove all personally identifiable information before sharing data with universities and/or research institutions.

## Appendix A

**Table A1:** Inter-rater reliability between raters based on Gwet’s AC1.

Behaviour Reported	Gwet’s AC1 Coefficient	Gwet’s AC1 Standard Error	Gwet’s AC1 Confidence Interval
Barking	0.853	0.031	0.792–0.913
Jumping up	0.824	0.033	0.759–0.889
Pulling on the lead	0.936	0.019	0.898–0.973
Recall issues	0.939	0.018	0.904–0.975
Destructive behaviour	0.944	0.017	0.911–0.977
Resource guarding	0.965	0.012	0.942–0.988
Toileting	0.958	0.014	0.931–0.985
Eating non-food items	0.971	0.011	0.949–0.993
All biting-type behaviours	0.993	0.005	0.983–1.000
Lunging	1.000	0.000	1.000–1.000
Growling and/or snarling	1.000	0.000	1.000–1.000
Chasing	0.941	0.017	0.907–0.974
Humping	0.972	0.011	0.951–0.993
Stealing	0.975	0.010	0.955–0.996
Scavenging	0.970	0.011	0.948–0.991
Separation-related behaviours	0.973	0.010	0.953–0.994
Displaying fear	0.884	0.024	0.836–0.933
Displaying agonistic behaviours	0.989	0.007	0.976–1.000
Displaying reactive behaviours	0.977	0.010	0.958–0.996
